# Nanosensors and particles: a technology frontier with pitfalls

**DOI:** 10.1186/s12951-019-0542-7

**Published:** 2019-10-28

**Authors:** Viola Vogel

**Affiliations:** Laboratory of Applied Mechanobiology, Department of Health Sciences and Technology, ETH Zurich, Vladimir-Prelog Weg 4 (HCI F443 Hönggerberg), 8093 Zurich, Switzerland

## Abstract

As we are approaching 20 years after the US National Nanotechnology Initiative has been announced, whereby most of that funding was spend to engineer, characterize and bring nanoparticles and nanosensors to the market, it is timely to assess the progress made. Beyond revolutionizing nonmedical applications, including construction materials and the food industry, as well as in vitro medical diagnostics, the progress in bringing them into the clinic has been far slower than expected. Even though most of the advances in nanosensor and nanoparticle research and development have been paid for by disease-oriented funding agencies, much of the gained knowledge can now be applied to treat or learn more about our environment, including water, soil, microbes and plants. As the amount of engineered nanoparticles that enter our environment is currently exponentially increasing, much tighter attention needs to be paid to assessing their health risk. This is urgent as the asbestos story told us important lessons how financial interests arising from a rapid build up of a flourishing industry has blocked and is still preventing a worldwide ban on asbestos, nearly 100 years after the first health risks were reported.

## Assessing the progress made

Life evolved highly integrated biological nanosensors for a large range of applications, including to store and compute information, to sense the metabolic activities to ensure steady energy supply as well as to sense and respond to a broad range of environmental stimuli and threads. Such nanosensors include enzymes, antibodies, DNA, photochromic systems and many others whose functions and mechanisms, by which they often convert energy, are still to be deciphered. In fact, the diversity found in microorganisms, plants and animals is so huge that atomistic insights into how these machineries work is not only academically intriguing, but has inspired already a diversity of new nanoscale designs.

Our ability to engineer nanosystems with tightly tailored functions has made rapid progress since nanotech tools became available to synthesize, visualize and characterize such systems. While the public often relates the term nanosensors with nanoparticles, the definition of nanosensors is much broader and includes all nanodevices that respond to physical or chemical stimuli and convert those into detectable signals. Engineered nanoparticles and nanosensors have been made from inorganic or organic, from synthetic or biological materials. Their specificity to probe environmental or biomedical processes can be greatly enhanced by functionalizing them with biomolecules, for example in ways that molecular recognition events will cause detectable physical changes.

This Commentary forms part of a special issue, dedicated to “Nanosensors” as we approach 20 years of announcing that major funding will be poured into the advancement of nanotechnology, first by the US National Nanotechnology Initiative (NNI) [1], followed closely by others in Europe and Asia. The key promises driving such significant investments into the development of a new generation of nanoparticles and nano scale sensors was their anticipated low cost in production, their specificity to target biomolecules, microbial cells and tissues, as well as to detect toxins. This opened the door to a range of medical applications, including transformative technologies for point of care monitoring and diagnostics devices. It’s thus a timely occasion to review the successes of nanoparticles and sensors tailored to serve highly specific functions, from medical applications [2–6] to sensing the environment [7–12], as well as to ask where and when caution is warranted [13–23].

Even though most of the advances in nanosensor and nanoparticle research and development have been paid for by funding agencies in the context of early detection and treatment of human diseases, much of the gained knowledge applies to natural nanoparticles as well, or can now be applied to learn more about our environment. It is thereby interesting to note that the worldwide budgets of agencies that focused on nanotechnologies in the context of biomedical sciences addressing diseases are magnitudes higher than those dedicated to analyze their risks and to protect our environment. Yet, many insights and developments in biomedicine can be translated to addressing environmental challenges. For example, the development of nanoparticles for diagnostic and therapeutic applications gave much insights into the plethora of schemes by which nanoparticles and sensors can be designed and furbished with specific functions, and how they need to be designed to allow them to pass major barriers of our bodies such as the skin, lung and intestine epithelia, or the blood–brain or blood–tissue barrier. Much has also been learned regarding the pharmacokinetics of nanosystems once applied to the skin, swallowed, injected or inhaled [6, 24]. While nanosensors have already revolutionized nonmedical applications, including construction materials and the food industry, as well as the diagnostic medtech market, i.e. the use of sensors for in vitro diagnostics [10, 11], the progress in bringing nanoparticles into the clinic has been far slower than expected. Even though the majority of nanotechnology funding in bioengineering and medicine went into approaches to target tumor tissues with nanoparticles, a thorough meta-analysis of the literature from the last decade revealed that only a tiny fraction of the administered nanoparticles (< 1%) were actually delivered to solid tumours, whether based on organic or inorganic materials and with just minor differences based on their physical characteristics [25].

## A thorough assessment of potential health risks is urgently needed

The largest production of nanoparticles today, however, is not for biomedical or sensory applications, but to enhance material properties, for agricultural applications [12, 26, 27], in the food industry [9, 12, 15–19, 22, 26, 27] and in cosmetics [20–22]. For example, silver, ZnO and CuO nanoparticles are increasingly used as biocides [8, 9, 13, 16, 18–20, 28–30]. There is however increasing evidence of their threat to “non-target” organisms [8, 9, 13, 16, 18–20, 28]. After the annual production of engineered nanoparticles increased from less than 60 tons worldwide in 2005 to an estimated 1000-fold today [21, 28, 29], many of them are released into our environment and make their way back into the food chain. This already leads to significant daily uptakes in plants [9, 12, 27, 31], animals and humans [15, 22, 23, 28, 29, 32]. The asbestos story told us important lessons how financial interests arising from a rapid build up of a flourishing industry around a material with remarkable technical properties. Economic interests have blocked and are still preventing a worldwide ban on asbestos, even today [33]. This is happening despite early warnings of severe health risks that go back to the 1920s, and broad recognition that asbestos causes cancer in the 1960s [34, 35]. In Switzerland, for example, the Schweizerische Unfallversicherungsanstalt (SUVA) recognized asbestosis as profession-related disease in 1939, yet the usage of asbestos was banned in Switzerland only in 1995. This illustrates that major investments into studying the health risks of nanoparticles and sensors that enter the environment or the human body in high quantities, most prominently via the food chain or the air are urgently needed [14, 15, 23].
